# Controlling taxa abundance improves metatranscriptomics differential analysis

**DOI:** 10.1186/s12866-023-02799-9

**Published:** 2023-03-07

**Authors:** Zhicheng Ji, Li Ma

**Affiliations:** 1 Department of Biostatistics and Bioinformatics, Duke University, Durham, USA; 2 Department of Statistical Science, Duke University, Durham, USA

**Keywords:** Metatranscriptomics, Shotgun sequencing, Microbiome, Differential analysis

## Abstract

**Background:**

A common task in analyzing metatranscriptomics data is to identify microbial metabolic pathways with differential RNA abundances across multiple sample groups. With information from paired metagenomics data, some differential methods control for either DNA or taxa abundances to address their strong correlation with RNA abundance. However, it remains unknown if both factors need to be controlled for simultaneously.

**Results:**

We discovered that when either DNA or taxa abundance is controlled for, RNA abundance still has a strong partial correlation with the other factor. In both simulation studies and a real data analysis, we demonstrated that controlling for both DNA and taxa abundances leads to superior performance compared to only controlling for one factor.

**Conclusions:**

To fully address the confounding effects in analyzing metatranscriptomics data, both DNA and taxa abundances need to be controlled for in the differential analysis.

**Supplementary Information:**

The online version contains supplementary material available at 10.1186/s12866-023-02799-9.

## Background

Metatranscriptomics [1] and metagenomics [2] studies by shotgun sequencing profile taxonomic abundances and RNA and DNA abundances of genes and gene pathways in a microbial community. They provide a more comprehensive landscape of microbial functions and activities than traditional technologies such as 16S rRNA sequencing [3] that only profile taxonomic abundances. Metatranscriptomics and metagenomics profiles of hundreds of individuals have been generated in large consortium studies such as The Integrative Human Microbiome Project (iHMP) [4]. A fundamental goal in analyzing such data is to understand how changes in microbial compositions or gene activities are associated with disease status, thus providing new insights into the disease mechanisms. This involves the comparisons of RNA, DNA, and taxa abundances across samples and the identification of pathways with differential RNA abundances between sample groups.

While significant progress has been made to develop methods for differential compositional analysis [5], methods to identify differentially expressed (DE) gene pathways for metatranscriptomics data are still underdeveloped. DE analysis for metatranscriptomics data is different from that for single-organism RNA-seq data in that the RNA abundance is highly related to the taxonomic abundances and genes’ copy numbers in a microbial community with many organisms. If DE analysis is directly performed without addressing these effects, it is hard to differentiate if the DE is due to the changes in DNA (metagenomic gene abundances) or taxonomic abundances or due to the changes in relative RNA abundances independent of DNA or taxonomic changes. Thus, methods designed for single-organism RNA-seq data such as limma [6], DESeq2 [7], and edgeR [8] cannot be directly applied. To address this issue, Biobakery 3 [9] takes advantage of the paired metagenomics information measured in the same individuals, and include DNA abundance as a covariate in a linear-mixed effect model to address the strong correlation between DNA and RNA abundance. A recent study [10] benchmarks the performance of six DE models controlling for either DNA or taxonomic abundances, and concludes that the model that only controls for DNA abundance has the preferred performance.

While the existing methods only control for DNA or taxonomic abundances when performing the differential analysis, it remains unknown if controlling for a single effect is sufficient to fully address the confounding effect, and if controlling for both DNA and taxonomic abundances will further improve the performance of the differential models.

## Results

**Fig. 1:**
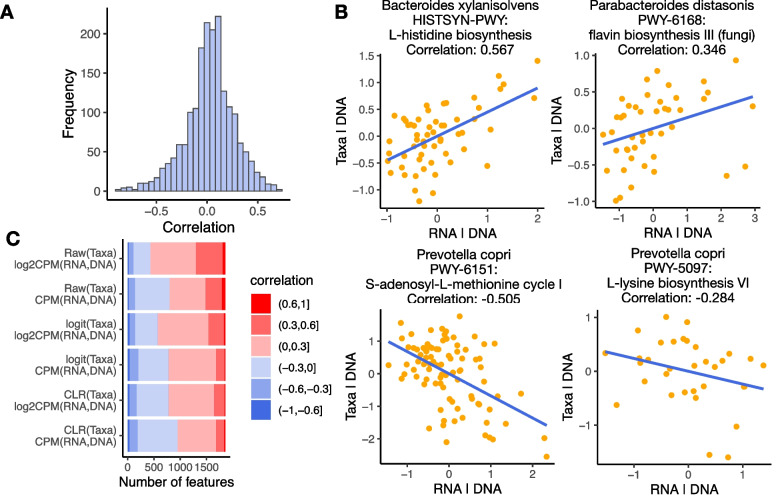
Partial correlations between RNA and taxonomic abundances controlling for DNA abundances **A** Distribution of partial correlations between RNA and taxonomic abundances controlling for DNA abundances. **B** Example features with positive or negative partial correlations between RNA and taxonomic abundances controlling for DNA abundances. x-axis shows the RNA abundances regressing out DNA abundances. y-axis shows the taxonomic abundances regressing out DNA abundances. **C** Number of features with partial correlations falling in different ranges. Each row represents one way of data transformation. Raw: raw proportion. logit: logit transformation. CLR: centered log-ratio transformation. CPM: count per million

**Fig. 2:**
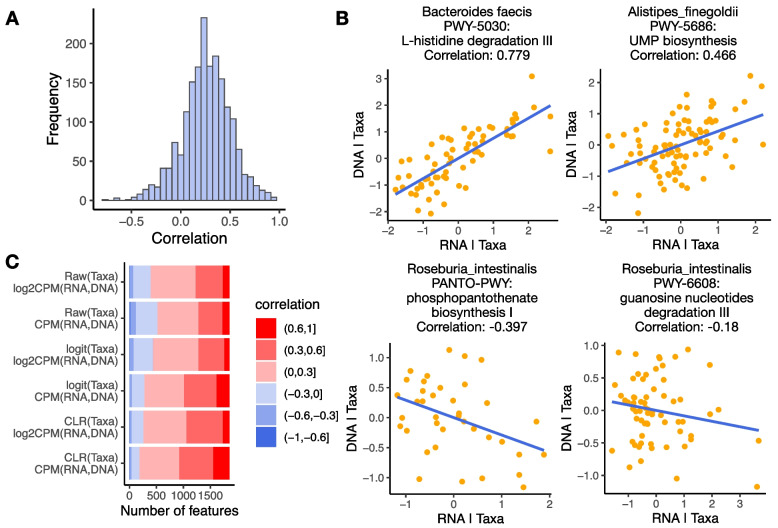
Partial correlations between RNA and DNA abundances controlling for taxonomic abundances **A** Distribution of partial correlations between RNA and DNA abundances controlling for taxonomic abundances. **B** Example features with positive or negative partial correlations between RNA and DNA abundances controlling for taxonomic abundances. x-axis shows the RNA abundances regressing out taxonomic abundances. y-axis shows the DNA abundances regressing out taxonomic abundances. **C** Number of features with partial correlations falling in different ranges. Each row represents one way of data transformation. Raw: raw proportion. logit: logit transformation. CLR: centered log-ratio transformation. CPM: count per million

The existing methods assume that including either DNA or taxonomic abundance in DE is sufficient to control for all confounding effects. Surprisingly, we found that if only DNA (metagenomic gene abundances) or taxonomic abundance is controlled for, there is still a strong partial correlation between RNA abundance with the other factor, meaning that the existing methods do not fully control for the confounding effects. For example, we downloaded and processed paired metatranscriptomics and metagenomics data from IBDMDB [11]. For each feature (gene pathway in a species), we calculated the partial correlation between RNA abundances (log2-transformed counts per million, CPM) and taxonomic abundances (centered log-ratio transformed, CLR) after controlling for DNA abundances (log2-transformed CPM). There are 9.1% of all features with partial correlations $< -0.3$ and 11.4% of all features with partial correlations $> 0.3$ (Fig. 1A-B). We also experimented with different ways of transforming RNA and DNA abundances (raw CPM or log2-transformed CPM), and transforming taxonomic abundances (CLR, logit, or raw proportion). There are similar numbers of features with high absolute values of partial correlations when data were transformed in different ways (Fig. 1C), suggesting that the partial correlations are unlikely to be only induced by how data were transformed. Likewise, we calculated the partial correlation between RNA abundances and DNA abundances (log2-transformed CPM) after controlling for taxonomic abundances (CLR transformed). There are 2.0% of all features with partial correlations $< -0.3$ and 43.2% of all features with partial correlations $> 0.3$ (Fig. 2A-B). There are again similar numbers of features with high absolute values of partial correlations when data were transformed in different ways (Fig. 2C). In both cases, we observe a large number of features with strong positive or negative partial correlations, suggesting that both DNA and taxonomic abundances need to be adjusted in DE to fully control for confounding effects. A high partial correlation between RNA and DNA abundances after controlling for taxonomic abundances is expected since RNA is a direct product of DNA thus RNA abundances are more directly associated with DNA abundances. A non-zero partial correlation between RNA and taxonomic abundances after controlling for DNA abundances is somewhat surprising. One potential reason is that the RNA abundances of a gene are not only decided by that gene’s DNA abundances, but also affected by interactions and communications with other microbes in the environment. A large number of microbes belonging to the same taxon may stimulate or inhibit the production of certain types of RNA to reach a state of equilibrium, bypassing the DNA abundances. Another potential reason is that the highly sparse and noisy DNA abundances measured by current sequencing technology may not be reliable enough to reflect the actual DNA abundances. The more robust measurements of taxonomic abundances carry additional information that may explain changes in RNA abundances. Diversity in physical characteristics across the microbes may also contribute to differences in gene transcription rate given the same amount of DNA content.

**Fig. 3:**
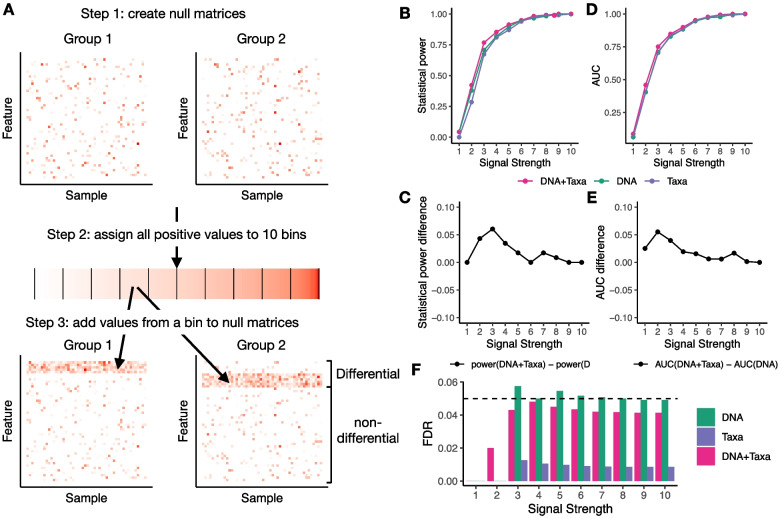
Simulation study 1. **A** Diagram showing the procedure of constructing simulation data. **B** Statistical power at 0.05 nominal FDR for different signal strengths. **C** Differences of statistical power between DNA+Taxa model and DNA only model. **D** AUC for different signal strengths. **E** Differences of AUC between DNA+Taxa model and DNA only model. **F** Real FDR (y-axis) of different methods with nominal FDR of 0.05 for different signal strengths

We conducted two simulation studies to systematically evaluate the performance of DE models when both or only one factor is controlled for. In the first study, the simulation dataset was constructed using real RNA, DNA (metagenomic gene abundances), and taxonomic abundance information from IBDMDB (Fig. 3A, Methods). We first created a null scenario where samples were randomly assigned to two groups and no differential signal was expected. We then sorted all positive values of RNA abundances in increasing order and assigned them to 10 bins. Finally we added the positive numbers from a bin to the RNA abundances of the null matrices to create differential signals. The differential signals were created only for pathways with high absolute values of partial correlations either between RNA and DNA abundances controlling taxonomic abundances or between RNA and taxonomic abundances controlling for DNA abundances. The 10 bins correspond to increasing levels of differential signals (Supplementary Fig. 1), with averaged absolute values of log2 fold change ranging from 0.341 (signal strength of 1) to 4.731 (signal strength of 10). Since the data has a longitudinal design, linear-mixed effect models were used for DE analysis (Methods). Three different models were applied for DE analysis: controlling for both DNA and taxonomic abundances (DNA+Taxa), controlling only for DNA abundances (DNA), and controlling only for taxonomic abundances (Taxa). We evaluated the performance of three models by statistical power at 0.05 FDR (Fig. 3B-C), and area under FDR-sensitivity curve (AUC, Fig. 3D-E). The model that controls for both factors (DNA+Taxa) has consistently the best performance in all scenarios. The DNA+Taxa model improves AUC by 0.055 over the DNA only model with signal strength of 2, representing a 13.7% increase of performance (Fig. 3E). Both DNA+Taxa model and DNA only model outperform the Taxa only model in statistical power, and DNA only model has comparable performance as Taxa only model in AUC. Both DNA+Taxa and Taxa only models are able to control false positives, while DNA only model slightly fails to control false positives in some cases (Fig. 3F).

**Fig. 4:**
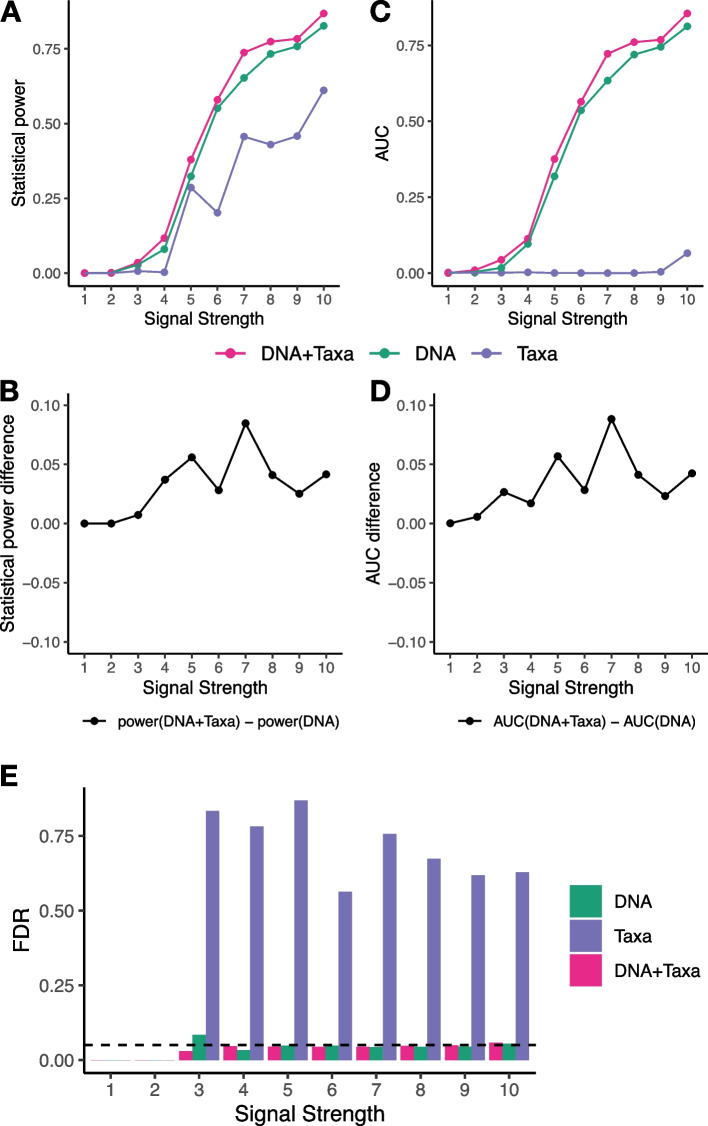
Simulation study 2 true-combo-bug-exp scenario. **A** Statistical power at 0.05 nominal FDR for different signal strengths. **B** Differences of statistical power between DNA+Taxa model and DNA only model. **C** AUC for different signal strengths. **D** Differences of AUC between DNA+Taxa model and DNA only model. **E** Real FDR (y-axis) of different methods with nominal FDR of 0.05 for different signal strengths

In the second study, we utilized a simulation strategy implemented in a previous study [10]. Four simulation scenarios were considered. In each scenario, samples were randomly split into two sample groups and differential analyses were performed between the two groups. In the first scenario, RNA abundances are associated with sample group identities for DE genes (true-exp). In the second scenario, both RNA abundances and taxonomic abundances are associated with sample group identities for DE genes (true-combo-bug-exp). In the third scenario, both RNA abundances and sequencing depths are associated with sample group identities for DE genes (true-combo-dep-exp). In the fourth scenario, RNA abundances of DE gene families are associated with sample group identities (group-true-exp). We tested the performance of DNA+Taxa, DNA, and Taxa models in all four scenarios. Ordinary linear regression was applied since there is no longitudinal design for this dataset. We find that the DNA+Taxa model significantly improves statistical power (Fig. 4A-B) and AUC (Fig. 4C-D) over DNA only model in the true-combo-bug-exp scenario, with the improvements almost reaching 0.1 in some cases. DNA+Taxa model has almost identical statistical power and AUC as DNA only model in other three scenarios (Supplementary Fig. 2). This is expected since only the true-combo-bug-exp scenario creates associations between taxa abundances and sample phenotypes. In all scenarios, DNA+Taxa model and DNA only model have significantly higher statistical power and AUC than Taxa only model. Taxa only model has close to zero statistical power and AUC when there is low signal strength in all scenarios. Taxa only model fails to control false positives in the true-combo-bug-exp scenario (Fig. 4E), while DNA+Taxa and DNA only models are able to control false positives in almost all cases (Fig. 4E, Supplementary Fig. 2).

**Fig. 5:**
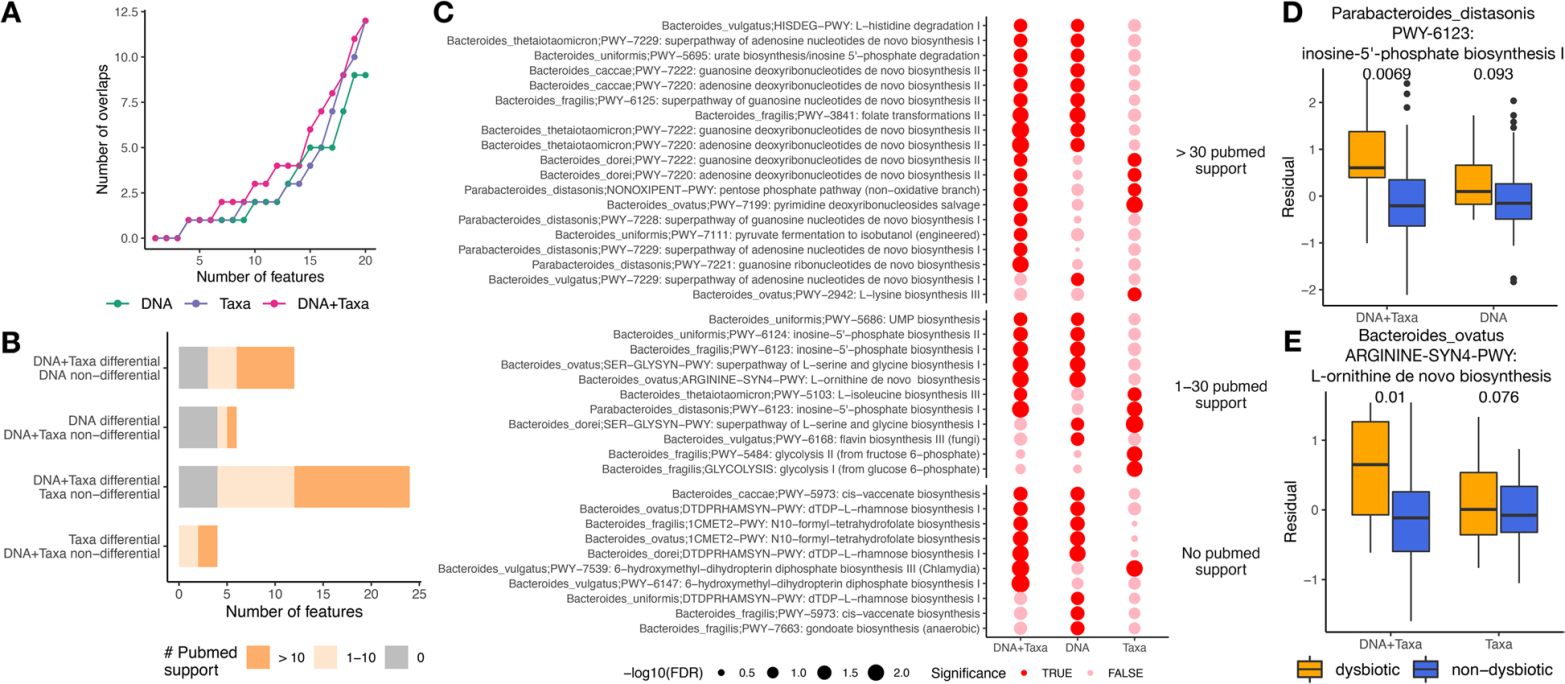
IBDMDB data analysis. **A** Reproducibility analysis. x-axis shows the number of top differential features. y-axis shows the number of top differential features identified in both subject groups. **B** Comparison of number of differential features identified by three DE models. Colors indicate the number of PubMed search hits for each differential feature. **C** Full list of differential features identified by at least one but not all DE models. Features with different levels of PubMed literature support are placed in three groups. **D** Example feature identified as differential by DNA+Taxa model but not by DNA model. y-axis shows the RNA abundances regressing out DNA and Taxa (left) or DNA (right), as well as covariates of age and antibiotic status. FDRs of statistical tests are indicated on the top. **E** Example feature identified as differential by DNA+Taxa model but not by Taxa model. y-axis shows the RNA abundances regressing out DNA and Taxa (left) or Taxa (right), as well as covariates of age and antibiotic status. FDRs of statistical tests are indicated on the top

As a real data example, we applied the three DE models (Methods) to the IBDMDB dataset to identify differential features between active (dysbiotic) and inactive (non-dysbiotic) time points in patients with Crohn’s disease (CD). We first performed a reproducibility analysis where all subjects (one subject may have multiple longitudinal samples) were randomly split into two groups and the three DE models were applied to each subject group. We evaluated how many top differential features can be reproducibly identified in both subject groups. Results identified by the model controlling for both factors (DNA+Taxa) are consistently more reproducible compared to results from other two models (Fig. 5A). Since the real biological signal is more likely to reproduce itself across multiple sample groups, this result suggests that the DNA+Taxa model is more capable of identifying real biological signals. We then applied the three DE models to the full dataset. Since a rigorous gold standard is lacking, we consulted PubMed and recorded numbers of publications supporting the differential features identified by each DE model (Supplementary Table 1, Methods). DNA+Taxa model identifies 12 differential features that cannot be identified by DNA model, and 9 of them have PubMed supports (Fig. 5B). DNA+Taxa model also identifies 24 differential features that cannot be identified by Taxa model, and 20 of them have PubMed supports (Fig. 5B). In comparison, DNA model identifies 6 differential features that cannot be identified by DNA+Taxa model, and 2 of them have PubMed supports (Fig. 5B). Taxa model identifies 4 differential features that cannot be identified by DNA+Taxa model, and 4 of them have PubMed supports (Fig. 5B). These results suggest that DNA+Taxa model is able to identify more differential features, and many of those new features are supported by existing literature. Figure 5C shows the names and differential status of features that are identified as differential in at least one but not all DE models. Figure 5D and E show two example features which are identified as significantly differential by DNA+Taxa model but non-significant by DNA or Taxa model. In both cases, RNA abundances controlled for both DNA and taxonomic abundances show much stronger differences between two sample groups compared to those when only one factor is controlled for. *Parabacteroides distasonis* was reported to have unclear mechanistic associations with Crohn’s disease [12], and inosine-5’-monophosphate dehydrogenase activities were found to be linked with metabolite ratios in patients with inflammatory bowel disease [13]. *Bacteroides ovatus* was reported to cause serum antibody responses in IBD patients [14], and the expression of Arginase 1 (Arg1), which converts L-arginine into ornithine and urea, correlates with the degree of inflammation in intestinal tissues from IBD patients [15]. These studies provide likely clues that the new features identified by DNA+Taxa model could generate relevant hypotheses to explore the mechanisms of CD and IBD.

## Discussion

Metatranscriptomics data contain information about multiple organisms, and the observed gene expression levels are highly correlated with DNA (metagenomic gene abundances) or taxonomic abundances. When performing differential gene expression analysis, existing methods control for either DNA or taxonomic abundances. This study demonstrates that controlling for a single factor is insufficient to fully address the confounding effect. Both factors need to be accounted for simultaneously in the differential model.

There are several limitations of this study. First, the analysis depends on samples with paired metatranscriptomics and metagenomics information. It is unclear how to adequately address the confounding issues if only metatranscriptomics information is available, and methods to accurately estimate DNA or taxonomic abundances from metatranscriptomics data are needed. Second, this study only discusses the effect of controlling for confounding effects in the gene expression differential analysis. The effect of controlling for confounding effects in other types of analysis remains unknown and is worth future explorations. Third, all functions of interest need to be assigned to specific taxa, which is not always possible especially in environments not well characterized.

## Conclusions

We reported significant partial correlations between RNA abundances and DNA or taxonomic abundance when the other factor is controlled for. We demonstrated that the DE model controlling for both DNA and taxonomic abundances outperforms DE models that only control for one factor in both simulation studies and real data analysis. This finding provides new insights for performing DE analysis or designing new DE methods for metatranscriptomics data.

## Methods

### IBDMDB data processing

RNA and DNA abundances of microbial metabolic pathways for each species, taxonomic abundances, and sample metadata of HMP2:IBDMDB dataset were downloaded from the IBDMDB website (https://ibdmdb.org/). The downloaded dataset contains the metagenomic and metatranscriptomic information of longitudinal samples collected from patients with CD and UC. The datasets were already processed by bioBakery Version 3.0 [9]. Unmapped reads or unknown taxa were removed from the data. Downloaded RNA and DNA abundances are in cpm (counts per million), and they were further log2 transformed after adding a pseudocount of 1. Taxa abundance values were centered log-ratio (CLR) transformed. Taxon with zero abundances were removed prior to CLR.

For each feature (gene pathway in a species), only samples that have all positive values in RNA, DNA, and taxonomic abundances are kept. Such filtering addresses the excessive amounts of zeroes in RNA abundances [9], and similar approach has been shown to have superior performance [10].

### Generating data for the first simulation study

Simulation datasets were created using the processed IBDMDB data (log2 transformed CPM values for DNA and RNA abundances, and CLR transformed taxonomic abundances). Figure 3A shows the schematic of generating the simulated data. All samples were randomly assigned to two groups with equal chances to create a null situation where no differential feature is expected between the two groups. Then, artificial differential signals were added to selected features with absolute values of partial correlations (either between RNA and DNA abundances controlling taxonomic abundances or between RNA and taxonomic abundances controlling DNA abundances) larger than 0.3. Specifically, all positive RNA abundance values across all samples and features are sorted and assigned to 10 bins, so that the minimum value in the *k*th bin is no smaller than the maximum value in the $(k-1)$th bin. The 10 bins represent different levels of differential signals. The first bin has the weakest differential signals and the last bin has the strongest differential signals. To add differential signals from *k*th bin to one feature, one of the two sample groups is randomly chosen to receive the differential signals, and RNA abundance values of all samples in the chosen sample group are added by values randomly selected from the *k*th bin. Differential features are then identified by each DE model and compared to the gold standard differential features. The simulation study is repeated for each choice of *k* ($k=1,2,...,10$). The injection of differential signals only causes negligible changes in total library size (Supplementary Fig. 3) so the library size was not recalibrated for the simulated datasets.

### Generating data for the second simulation study

The simulation data were generated using the code in a Github repository (https://github.com/biobakery/MTX_synthetic) that implements the same procedures described in a previous study [10]. In true-exp mode, the data were generated with parameters –spike-exp 0.1 –spike-exp-strength j. In true-combo-dep-exp mode, the data were generated with parameters –spike-dep –spike-exp 0.1 –spike-exp-strength j. In true-combo-bug-exp mode, the data were generated with parameters –spike-bug 0.5 –spike-exp 0.1 –spike-exp-strength j. In group-true-exp mode, the data were generated with parameters –spike-groups –spike-exp 0.1 –spike-exp-strength j. Here j controls the signal strengths and takes values 0.1, 0.2, ..., 1. The weakest signal strength of 1 corresponds to j being 0.1, and the strongest signal strength of 10 corresponds to j being 1. The values of all other parameters were the same as the values used in the original Github repository.

### Statistical models

The HMP2:IBDMDB study provides longitudinal measurements for each subject. After data filtering described in the “IBDMDB data processing” section, we observed in some features that most subjects only have one longitudinal observation, while in other features many subjects have multiple longitudinal observations. Thus we first used a model selection procedure to determine if a linear model with only fixed effects or a linear-mixed effect model is needed for each feature. The two models differ in that the linear-mixed effect model includes a random effect indicating which subject each longitudinal observation was collected. For each feature we fitted both models and compared their model fittings using a likelihood ratio test. Throughout the study, the linear-mixed effect model was fitted using lmerTest::lmer() function in R and the ordinary linear model was fitted using stats::lm() function in R. The p-values were obtained with asymptotic chi-squared tests (lmerTest::anova() function in R) and adjusted by BH procedure [16] (stats::p.adjust() function in R) to obtain FDRs. For all features with FDR < 0.05, linear-mixed effect models were used. For all features with FDR > 0.05, linear models with only fixed effects were used.

For simulation studies based on HMP2:IBDMDB data, the linear mixed-effect models are $RNA \sim DNA+Taxa+group+(1 \vert subject)$ for the DNA+Taxa model, $RNA \sim DNA+group+(1 \vert subject)$ for the DNA model, and $RNA \sim Taxa+group+(1 \vert subject)$ for the Taxa model. For model selection described above, their corresponding ordinary linear regression models are $RNA \sim DNA+Taxa+group$, $RNA \sim DNA+group$, and $RNA \sim Taxa+group$. Here *RNA*, *DNA*, and *Taxa* represent the processed RNA, DNA, and taxonomic abundances. *group* indicates the two sample groups being compared with, and *subject* indicates the subjects from which samples are longitudinally collected. The p-values of the group differences (*group*) are of primary interest and extracted from the model fitting (lmerTest::summary() function in R).

For real data analysis of HMP2:IBDMDB data, the linear mixed-effect models are $RNA \sim DNA+Taxa+active+age+antibiotics+(1 \vert subject)$ for the DNA+Taxa model, $RNA \sim DNA+active+age+antibiotics+(1 \vert subject)$ for the DNA model, and $RNA \sim Taxa+active+age+antibiotics+(1 \vert subject)$ for the Taxa model. For model selection described above, their corresponding ordinary linear regression models are $RNA \sim DNA+Taxa+active+age+antibiotics$, $RNA \sim DNA+active+age+antibiotics$, and $RNA \sim Taxa+active+age+antibiotics$. Here *active* indicates the dysbiotic and non-dysbiotic sample groups being compared with, and *age* and *antibiotics* are the age and antibiotics status for each subject, similar to a previous study [9]. The p-values of the differences between dysbiotic and non-dysbiotic sample groups (*active*) are of primary interest and extracted from the model fitting (lmerTest::summary() function in R).

For simulation studies based on a previous study [10], we used ordinary linear regression since there is no longitudinal design. The DNA+Taxa model is: $RNA \sim DNA+Taxa+group$. The DNA model is: $RNA \sim DNA+group$. The Taxa model is: $RNA \sim Taxa+group$. The p-values of the group differences (*group*) are of primary interest and extracted from the model fitting (stats::summary() function in R).

DE analysis was performed only in features with at least 10 observations in both sample groups after data filtering. To test for the fixed effect of interest, the Satterthwaite method [17] was used to estimate degrees of freedoms and p-values were obtained with t-tests. These procedures are already implemented in lmerTest::summary() function in R. FDRs were obtained using BH procedure [16] (stats::p.adjust() function in R).

### PubMed support search for real data analysis

For each feature, we manually identified a keyword of its associated metabolic gene pathway. We searched PubMed with the combination of “crohn’s disease” and the keyword. We additionally require that both keywords need to appear in either main text or title/abstract of a paper. For example, the PubMed search is “(“crohn disease”[Text Word] OR “crohn disease”[Title/Abstract]) AND (“adenosine”[Text Word] OR “adenosine”[Title/Abstract])” if the keyword is “adenosine”. We then recorded the number of publications found by PubMed. For each keyword with at least one Pubmed record, we performed a manual validation to identify one example publication that mentions both “crohn disease" and the keyword of the target feature. The name of the feature, the search keyword, the Pubmed search term, the number of Pubmed records, Pubmed ID of the manually validated publication, and the date the search was performed are all listed in Supplementary Table 1.

### Software used in this study

All data processing and statistical analysis were performed using R version 4.1.1. All figures were generated using ggplot2 [18] version 3.3.6. Linear mixed-effect models were performed using lmerTest version 3.1-3.

### Code availability

All code, scripts, and processed HMP2:IBDMDB data to reproduce the analysis in this study have been made freely available on Github: https://github.com/zji90/microbiome_taxacontrol. A static version of the code can be found on Zenodo: https://doi.org/10.5281/zenodo.7222142.

## Supplementary Information

**Additional file 1:** **Supplementary Figure 1.**  fold change of different signal strengths in simulation study 1. x-axis shows the signal strengths. y-axis shows the absolute value of log2 fold changes between sample groups being compared with.

**Additional file 2:** **Supplementary Figure 2.** . results of three scenarios for simulation study 2. The first column shows statistical power at 0.05 nominal FDR. The second column shows AUC. The third column shows the real FDR at 0.5 nominal FDR. **A** Results for group-true-exp scenario. **B** Results for true-combo-dep-exp scenario. **C** Results for true-exp scenario.

**Additional file 3:** **Supplementary Figure 3. **proportion changes in library size for simulation study 1. For each sample, proportion of changes in library size before and after the simulation signals are injected.

**Additional file 4:** **Supplementary Table 1.** list of differential features and PubMed literature support. 1st column: name of the feature; 2nd column: name of the gene pathway; 3rd column: keyword of the pathway used to perform literature search; 4th column: term used in Pubmed search; 5th column: Number of PubMed hits searching for both crohn's disease and the keyword in the third column. 6th-8th columns: If the feature is identified as differential in DNA+Taxa, DNA, or Taxa model. 9th column: Pubmed ID of the paper manually validated to be associated with both crohn's disease and the keyword in the third column. 10th column: date search was performed.

## Data Availability

The processed IBDMDB data was downloaded from the IBDMDB website https://ibdmdb.org/.

## Abbreviations

RNA: metatranscriptomic RNA abundance
DNA: metagenomic gene abundance
Taxa: taxonomic abundance
iHMP: The Integrative Human Microbiome Project
IBDMDB: The Inflammatory Bowel Disease Multi’omics Database
IBD: inflammatory bowel disease
DE: differentially expressed
CPM: counts per million
CLR: centered log-ratio transformation
AUC: area under FDR-sensitivity curve
FDR: false discovery rate
CD: crohn’s disease
