# Supplementary figures and images for: Controlling taxa abundance improves metatranscriptomics differential analysis
Source: BMC Microbiol. 2023 Mar 7;23:60. doi: 10.1186/s12866-023-02799-9 (PMC9990291; doi:10.1186/s12866-023-02799-9)

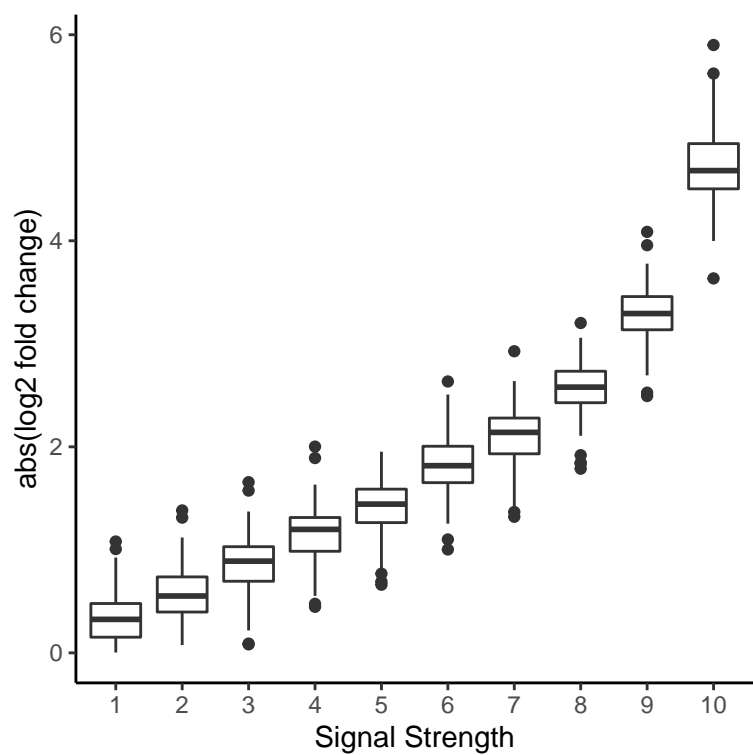

Supplement: Supplementary file 1 — Additional file 1: Supplementary Figure 1. fold change of different signal strengths in simulation study 1. x-axis shows the signal strengths. y-axis shows the absolute value of log2 fold changes between sample groups being compared with. [file 12866_2023_2799_MOESM1_ESM.pdf]

**A**

group-true-exp

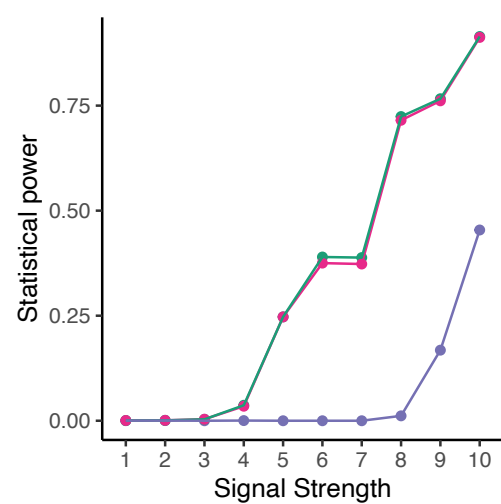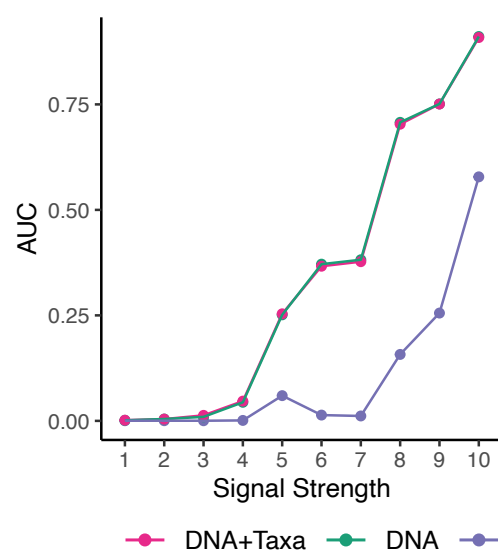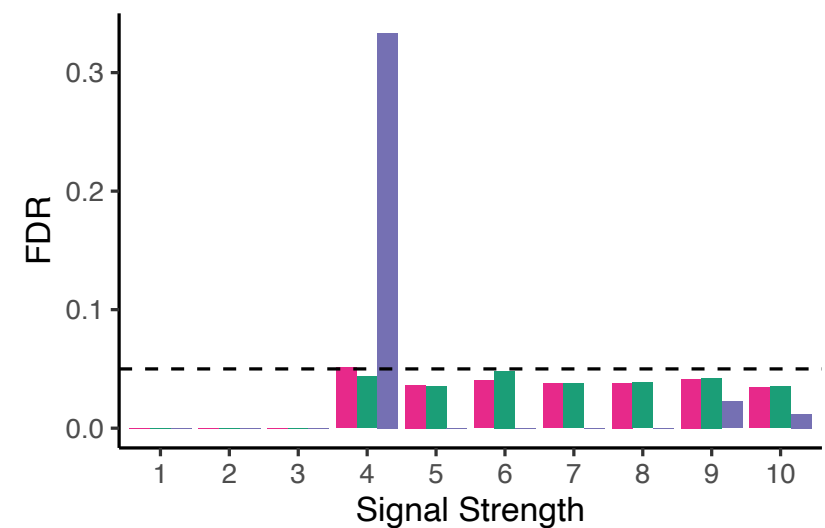**B**

true-combo-dep-exp

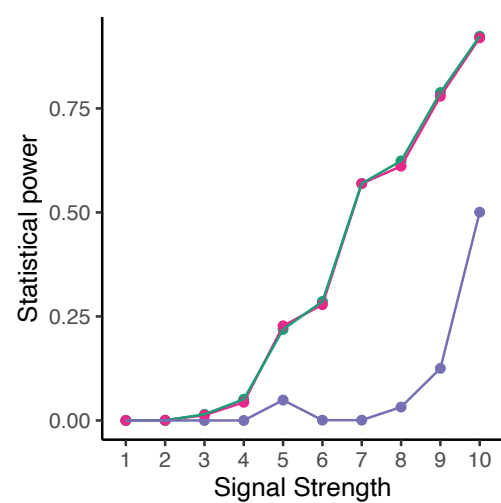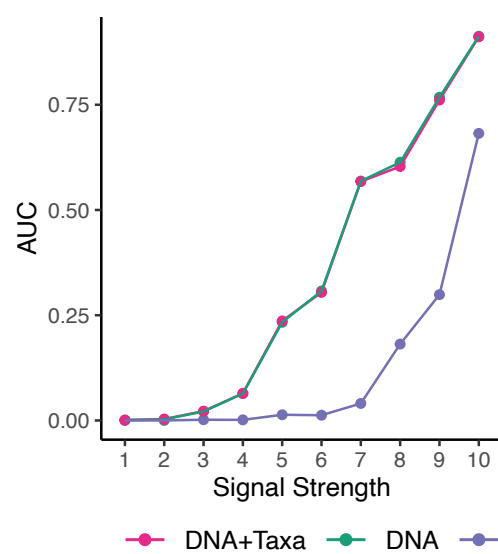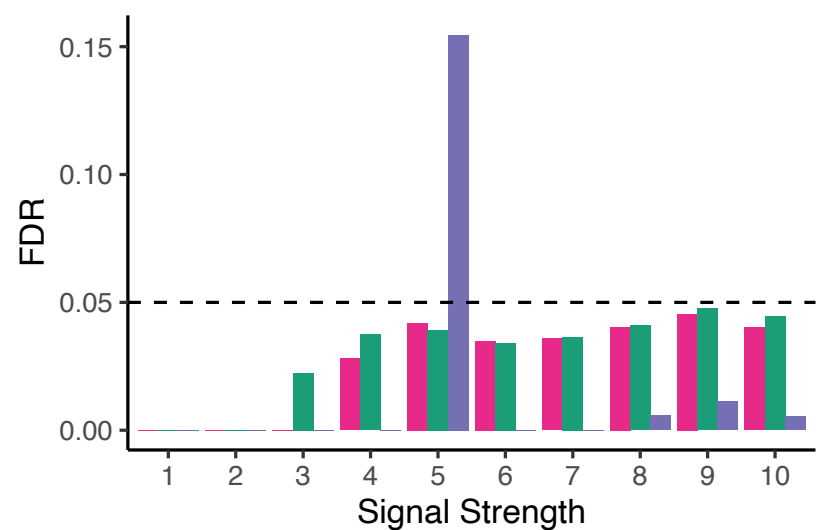**C**

true-exp

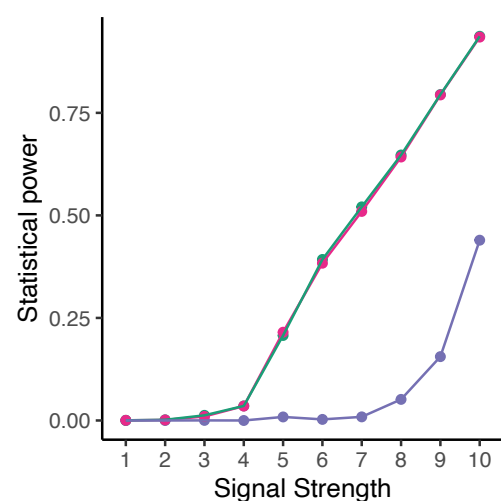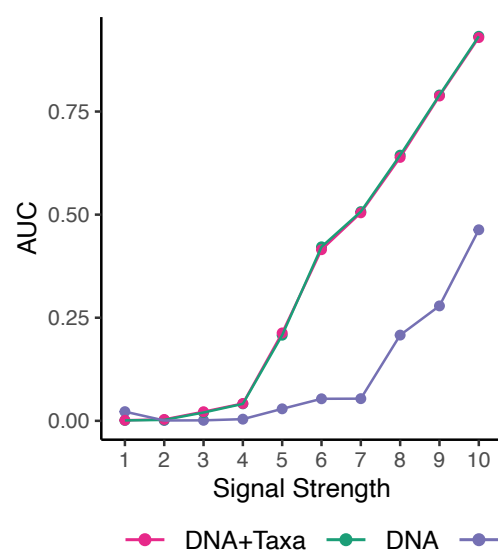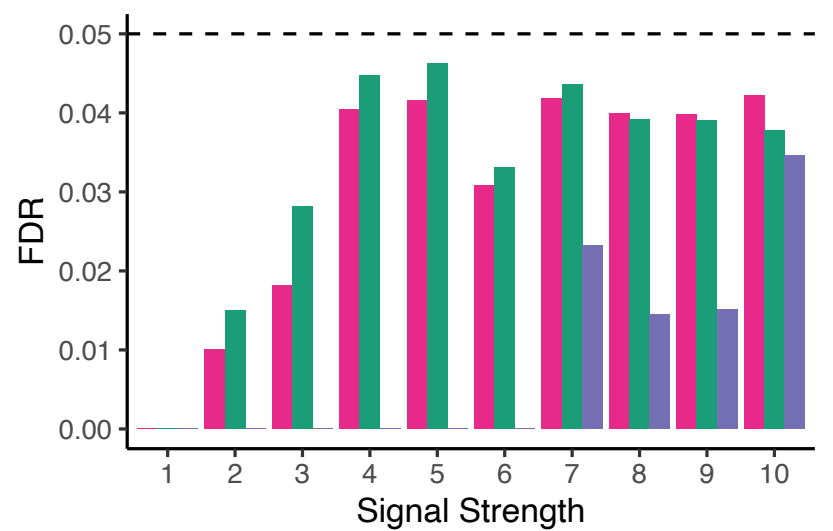

Supplement: Supplementary file 2 — Additional file 2: Supplementary Figure 2. . results of three scenarios for simulation study 2. The first column shows statistical power at 0.05 nominal FDR. The second column shows AUC. The third column shows the real FDR at 0.5 nominal FDR. A Results for group-true-exp scenario. B Results for true-combo-dep-exp scenario. C Results for true-exp scenario. [file 12866_2023_2799_MOESM2_ESM.pdf]

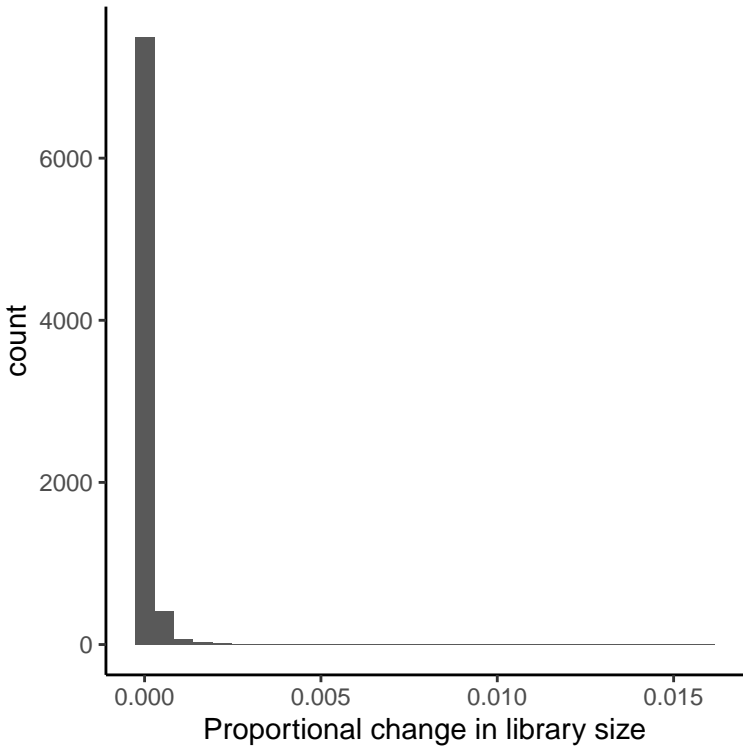

Supplement: Supplementary file 3 — Additional file 3: Supplementary Figure 3. proportion changes in library size for simulation study 1. For each sample, proportion of changes in library size before and after the simulation signals are injected. [file 12866_2023_2799_MOESM3_ESM.pdf]
